# A quality-adjusted survival analysis (Q-TWiST) of rituximab plus CVP *vs* CVP alone in first-line treatment of advanced follicular non-Hodgkin's lymphoma

**DOI:** 10.1038/sj.bjc.6605443

**Published:** 2009-11-17

**Authors:** R Marcus, R Aultman, F Jost

**Affiliations:** 1 Department of Haematology, Kings College Hospital, London, UK; 2 F. Hoffmann-La Roche Ltd, Basel, Switzerland

**Keywords:** follicular lymphoma, cyclophosphamide, vincristine, and prednisone, rituximab, quality-adjusted survival, health-related quality of life; quality-adjusted time without disease symptoms or toxicities of treatment

## Abstract

**Background:**

To evaluate the impact of treatment on health states that affect patients’ quality of life in advanced follicular lymphoma.

**Methods:**

A quality-adjusted time without symptoms of disease or toxicity of treatment (Q-TwiST) analysis was performed on data from a phase III clinical trial (Marcus *et al*, 2008).

**Results:**

Cyclophosphamide, vincristine, and prednisone plus rituximab (R-CVP)-treated patients gained a mean of 15.17 months in TWiST, 8.33 months in Q-TwiST, and 11.30 months less in disease relapse, without increase in toxicity compared with cyclophosphamide, vincristine, and prednisone (CVP)-treated patients.

**Conclusion:**

Rituximab plus CVP-treated patients reached a significant and clinically meaningful improvement within 12 months in quality-adjusted survival compared with CVP.

Follicular lymphoma (FL) is the most common form of non-Hodgkin's lymphoma (NHL), accounting for about 70% of indolent lymphomas and 20–25% of all cases of NHL (NHL classification project, 1997). Follicular lymphoma is characterised by slow disease progression and exhibits repeated chemotherapy-induced remissions followed by relapses, with times of remission and a median survival of 6–10 years (Horning, 1993), depending on the stage of disease and other prognostic variables at diagnosis. An open-label, randomised, multi-centre phase III trial investigated the clinical outcomes of previously untreated FL patients who received eight cycles of cyclophosphamide, vincristine, and prednisone (CVP) *vs* eight cycles of CVP plus rituximab (R-CVP) therapy (Marcus *et al*, 2005). The recently published 53-month median follow-up data showed that R-CVP-treated patients experienced clinically significant improvements in time to treatment failure and overall survival (OS), without an increase in clinically significant toxicity (Marcus *et al*, 2008). Although treatment goals for FL include freedom from symptomatic disease and toxicity-related impairment of quality of life (QoL), no QoL parameters were collected in this trial.

It is reasonable to assume that patients with no disease symptoms or treatment toxicity will have better health-related quality of life (HR-QoL) than those exhibiting disease symptoms and toxicity. Our objective was to conduct a quality-adjusted time without symptoms of disease or toxicity of treatment (Q-TWiST) analysis on the 53-month follow-up data from the phase III trial (Marcus *et al*, 2008). The Q-TWiST method was initially used to evaluate adjuvant therapies for breast cancer (Fairclough *et al*, 1999), and has since been applied to trials of interferon in advanced FL (Cole *et al*, 1998) and multiple myeloma (Zee *et al*, 1998).

## Materials and methods

### Patient population and treatment comparators

The design and main results of the phase III randomised, controlled clinical trial have been reported elsewhere (Marcus *et al*, 2005, 2008).

Analyses were conducted on all patients who received at least one administration of study medication. Patients were randomly assigned to R-CVP (*n*=162) or CVP alone (*n*=159). Patients treated with CVP alone received a combination of cyclophosphamide 750 mg m^−2^ i.v. on day 1; vincristine 1.4 mg m^−2^, up to a maximal dose of 2 mg i.v. on day 1; and prednisone 40 mg m^−2^ p.o. on days 1–5. Patients treated with R-CVP also received rituximab 375 mg m^−2^ i.v. on day 1 of each treatment cycle (length 21 days). Patients were treated for a maximum of eight cycles.

### Q-TWiST analysis

The Q-TWiST analysis was performed on the 321 patients and consisted of three steps (Staquet *et al*, 1998; Fayers and Machin, 2000).

#### Step 1: Definition of clinical health states

The clinical health states considered relevant to treatment decision-making in patients with FL were as follows: TOX – the time period with treatment-related adverse events; REL – the time period with disease relapse (progression), ending with death or censoring; and TWiST – the time period during which patients experienced no disease symptoms or treatment toxicities, thus reflecting the best possible patient HR-QoL in this clinical setting.

This Q-TWiST analysis was performed using all investigator-determined treatment-related adverse events occurring from the start of treatment until 28 days beyond the last protocol-defined dose or progression. Although there was a higher incidence of grade 3–4 neutropenia during treatment with R-CVP, this did not translate into a higher infection rate (Marcus *et al*, 2005, 2008).

#### Step 2: Estimation of health state duration

**Figure 1:**
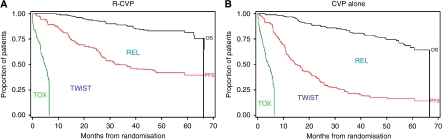
Partitioned survival plots for (**A**) R-CVP and (**B**) CVP alone. Curves represent overall survival (OS), progression-free survival (PFS), and treatment toxicity (TOX). Areas between the curves represent the mean time spent in the health states: TOX, time without disease symptoms or treatment toxicity (TWiST), and time in relapse (REL), based on 53 months’ median follow-up truncated at 67 months.

Mean duration of OS partitioned into health states TOX, TWiST, and REL was estimated from the phase III trial data (Marcus *et al*, 2008). Owing to the shorter follow-up in the CVP arm, the event data were truncated to 67 months, the longest follow-up in the shortest progression-free curve of the comparator, to exclude follow-up time bias in favour of R-CVP. The mean duration of toxicity was calculated without restriction, whereas the mean time spent in REL and TWiST was restricted to the clinical follow-up period. These estimates are represented by the area between the partitioned curves (Figure 1).

#### Step 3: Estimation of Q-TWiST

A quality-adjusted survival model was developed with the use of utility coefficients for *u*_TOX_, *u*_TWiST_, and *u*_REL_ to reflect the impact on the patient's HR-QoL. The utility coefficients are measured on a scale from 0 to 1, where 0 represents death and 1 represents the best possible patient QoL. Quality-adjusted time without disease symptoms or toxicities of treatment, defined as the weighted sum of time spent in each disease state is calculated as: 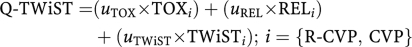


The utility weight of 0.618 (s.e., 0.056) used for REL was obtained from the UK study in 222 patients with FL (Pettengell *et al*, 2008). The base case Q-TWiST analysis assumed a TWiST utility of 1.0 and a utility of 0.618 for REL and TOX.

### Statistical analysis

Point estimates of mean differences in OS, progression-free survival (PFS), disease relapse (REL=OS–PFS), TWiST (TWiST=PFS–TOX), and duration of TOX were calculated from patient follow-up data. Owing to the unknown distributions of the mean differences, non-parametric bootstrapping, a numerical resampling method, was performed (5000 iterations) to obtain reliable estimates of the s.e. for the clinical end points (Fine and Gelber, 2001). *P*-values with 95% CI were reported on the basis of the standard *Z*-statistic.

### Threshold utility analysis

A threshold utility analysis assessed the Q-TWiST outcome over seven possible combinations of TOX and REL utility values with the TWiST utility set to 1.0 or 0.805 from the UK utility study (Pettengell *et al*, 2008).

### Probabilistic sensitivity analysis

Probabilistic sensitivity analysis (PSA) was performed to address the uncertainty of the clinical end points and the co-dependency between utilities and the health states. This was carried out using Monte Carlo simulations (1000 iterations), in which OS, PFS, REL, and TOX utilities were randomly sampled from Beta-Pert distributions (Vose, 2000), with the most likely and extreme values obtained from the mean and s.e. reported in the 53-month update (Marcus *et al*, 2008) and the UK utility study (Pettengell *et al*, 2008).

## Results

### Q-TWiST analysis

Survival times partitioned into the three health states are shown separately for R-CVP and CVP in Figure 1A and B, depicting the time spent in each health state over the follow-up period. Rituximab plus CVP-treated patients gained a mean of 15.17 months TWiST (*P*<0.001), and spent a mean of 11.30 months less time in relapse (*P*<0.001) compared with CVP patients, without any increase in toxicity (mean difference 0.24 months (*P*=0.36; Table 1). Using the patient-reported utility of 0.618 for REL, an assumed utility of 0.618 for TOX, and a utility of 1.0 for TWiST, R-CVP patients experienced a mean of 8.33 months’ longer Q-TWiST compared with CVP (95% CI 4.51–9.25, *P*<0.001) (Table 1). On the basis of PSA, the mean increase in Q-TWiST for R-CVP compared with CVP was 7.38 (95% CI: 5.82–10.87), which is consistent with the bootstrapped estimates.

### Utility threshold analysis

Results from the threshold utility analysis show that in all cases but one, the mean increases in Q-TWiST obtained with R-CVP *vs* CVP were statistically significant (Table 2).

### Gain function

**Figure 2:**
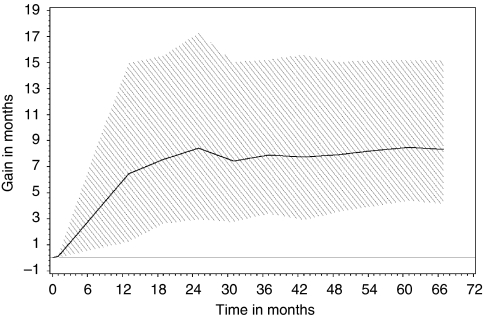
Gain function of incremental mean differences in Q-TWiST between R-CVP and CVP as a function of time (53 months’ median follow-up truncated at 67 months). The solid line represents the incremental mean differences in Q-TWiST over time with utilities 0.618 for TOX and REL and 1.0 for TWiST, whereas the shaded area depicts the range of differences in Q-TWiST, as the utility values for REL and TOX vary between 0 and 1.

The gain function (Staquet *et al*, 1998; Fayers and Machin, 2000), defined as the incremental mean differences in Q-TWiST between R-CVP and CVP over time, is shown in Figure 2.

## Discussion

### Comparative clinical and health outcomes

Long-term follow-up data (Marcus *et al*, 2008) showed statistically significant and clinically meaningful improvement for R-CVP over CVP in time to treatment failure and PFS, without an increase in clinically significant toxicity. Furthermore, OS for patients treated with R-CVP was significantly longer than for patients treated with CVP alone (Marcus *et al*, 2008). Quality-adjusted time without disease symptoms or toxicity of treatment analysis based on this 53-month update to the data shows that patients treated with R-CVP gained more time without treatment toxicities or disease symptoms and spent less time in relapse than did patients treated with CVP alone. Incorporating patient-reported utilities from the UK study in FL (Pettengell *et al*, 2008) confirmed the significant improvement in quality-adjusted survival with R-CVP *vs* CVP alone.

In their investigation of clinically important differences in Q-TWiST analyses, Revicki *et al* (2006) suggested that differences of 10–15% should be regarded as clinically important. A 15% increase in Q-TWiST was obtained with R-CVP *vs* CVP alone representing a clinically meaningful difference, with an 11% (mean 6.11 months) difference achieved within 12 months.

A previous Q-TWiST analysis comparing CHVP with CHVP plus IFN-*α*2b in 242 patients with FL (Cole *et al*, 1998) showed that after a median follow-up of 72 months, the IFN group gained a mean of 12.3 months’ PFS and 7.4 months’ OS, but experienced additional time with grade 3 or worse toxicity, compared with the CHVP group. In contrast, our study with 53 months’ median follow-up revealed that R-CVP treatment resulted in a mean increase in PFS of 15.4 months, a mean of 11.30 months’ less time spent in relapse, with no significant increase in toxicity *vs* CVP alone.

### Strengths and weaknesses

In the threshold utility analysis, the increase in Q-TWiST was not statistically significant with the utility combination 0.90, 0.10 and 0.805 for REL, TOX and TWiST respectively (Table 2). However, it is clinically unlikely that the QoL of FL patients who have relapsed would be better than that of patients without symptoms or toxicity.

The bootstrap analysis incorporated uncertainty in the clinical end points used to estimate Q-TWiST using deterministic utility values. Probabilistic sensitivity analysis, by addressing the relationship between OS, PFS, REL, TOX, and utilities, represents a comprehensive sensitivity analysis to test the robustness of the Q-TWiST outcome.

The UK utility study did not investigate patient utilities for TOX. However, as there was no significant difference in the duration of toxicity between the treatment arms, the Q-TWiST results were insensitive to this utility. Finally, a median 53-month follow-up period is relatively short given that patients with FL have a median survival of 6–10 years (Horning, 1993). Parametric extrapolation of the clinical data beyond this period is one approach for determining whether R-CVP will further augment quality-adjusted survival.

## Conclusions

Rituximab plus CVP-treated patients with advanced FL reached a significant and clinically meaningful improvement within 12 months in quality-adjusted survival compared with CVP alone.

## Conflict of interest

The study described was funded by F. Hoffmann-La Roche Ltd, Basel, Switzerland. R Aultman and F Jost are employees of F. Hoffmann-La Roche; R Marcus has provided *ad hoc* consulting services for F. Hoffmann-La Roche, and has received honoraria and attended paid Advisory Boards for F. Hoffmann-La Roche.

## Figures and Tables

**Table 1:** Components of Q-TWiST with utility values *u*_TOX_=0.618, *u*_REL_=0.618, and *u*_TWiST_=1.0

**End point^a^**	**Mean R–CVP (months)**	**Mean CVP (months)**	**Mean difference (months)**	**95% CI for mean difference^b^ (months)**	***P*-value**
OS	59.68	55.56	4.11	0.50 to 6.05	0.015
PFS	39.26	23.86	15.41	8.80 to 16.07	<0.001
TOX	4.12	3.88	0.24	−0.34 to 0.48	0.36
REL	20.41	31.71	−11.30	−13.42 to −6.21	<0.001
TWiST	35.15	19.98	15.17	8.65 to 16.06	<0.001
Q-TWiST	50.31	41.97	8.33	4.51 to 9.25	<0.001

Abbreviations: CI=confidence interval, OS=overall survival, PFS=progression-free survival, Q-TWiST=quality-adjusted time without symptoms of disease or toxicity, REL=disease relapse, TOX=toxicity.

a The estimated deterministic mean durations of the end points were restricted to 53 months median follow-up truncated at 67 months.

b 95% CIs were estimated using the non-parametric bootstrap method.

**Table 2:** Threshold utility analysis using three-way combinations of health state utility values to estimate Q-TWiST confidence intervals^a^

**TWiST utility**	**REL, TOX utilities**						
	0.1, 0.9	0.2, 0.8	0.4, 0.6	0.5, 0.5	0.6, 0.4	0.8, 0.2	0.9, 0.1
1.0	11.96	11.00	8.98	8.00	7.00	5.01	4.02
	(8.10–14.83)^*^	(7.42–13.79)^*^	(6.10–11.51)^*^	(5.43–10.50)^*^	(4.65–9.38)^*^	(2.72–7.55)^*^	(1.51–6.76)^*^
0.805	9.49	8.49	6.50	5.51	4.51	2.52	1.52
	(6.48–11.83)^*^	(5.93–10.85)^*^	(4.46–8.58)^*^	(3.60–7.51)^*^	(2.67–6.54)^*^	(0.43–4.91)^†^	(−0.70–4.03)
							NS

Abbreviations: Q-TWiST=quality-adjusted time without symptoms of disease or toxicity, REL=disease relapse, TOX=toxicity, NS=not statistically significant (*P*=0.124).

a Q-TWiST mean differences and associated 95% confidence intervals in parentheses were estimated using the non-parametric bootstrap method.

^*^P<0.001.

^†^*P*<0.05.
